# Lack of association of *TP73* rare variants with amyotrophic lateral sclerosis in a Chinese cohort

**DOI:** 10.1186/s40246-022-00437-5

**Published:** 2022-11-30

**Authors:** Chunyu Li, Yanbing Hou, Qianqian Wei, Junyu Lin, Qirui Jiang, Tianmi Yang, Yi Xiao, Jingxuan Huang, Yangfan Cheng, Ruwei Ou, Kuncheng Liu, Xueping Chen, Wei Song, Bi Zhao, Ying Wu, Bei Cao, Yongping Chen, Huifang Shang

**Affiliations:** Department of Neurology, Laboratory of Neurodegenerative Disorders, National Clinical Research Center for Geriatrics, West China Hospital, Sichuan University, No.37, Guoxue Lane, Chengdu, 610041 Sichuan China

**Keywords:** Amyotrophic lateral sclerosis, Rare variant, *TP73*

## Abstract

**Background:**

Recently, several rare variants of *TP73* were identified as potential disease cause for amyotrophic lateral sclerosis (ALS) in the European population. However, further replication was still necessary, especially in cohorts with different ethnic backgrounds.

**Methods:**

To explore the genetic role of *TP73* in ALS in the Asian population, we analyzed the rare protein-coding variants in 2011 patients with ALS and 3298 controls with whole-exome sequencing. Fisher’s exact test was performed between each variant and disease risk, while at gene level over-representation of rare variants in patients was examined with optimized sequence kernel association test.

**Results:**

Totally 24 rare variants with minor allele frequency < 0.01 were identified, among which nine were absent in controls. One variant p.P335T was previously reported, and another three variants were in the same amino acids as the variants reported in previous studies (p.R36Q, p.R414Q, p.R78C). At gene level, rare variants of *TP73* were not enriched in patients*.*

**Conclusions:**

Our findings did not support the genetic role of *TP73* in ALS in the Chinese population. Replication of specific variants identified in patients from different cohorts might provide additional insight. The current results also broadened the mutation spectrum of *TP73* and paved the way for further research.

**Supplementary Information:**

The online version contains supplementary material available at 10.1186/s40246-022-00437-5.

## Introduction

Amyotrophic lateral sclerosis (ALS) is a severe motor neuron disease characterized by the degeneration of upper and lower motor neurons [1]. Evidence from clinical and basic research suggested multiple causes of ALS, including the essential role of genetic components. Since the first ALS causative gene *SOD1* was identified in 1993, mutations in more than 50 genes have been identified as potential cause for ALS so far [2]. However, the majority of patients still have no identifiable genetic causes, indicating more risk genes are to be explored.

Recently, an exome sequencing study identified several rare heterozygous variants in the tumor suppressor gene *tumor protein p73* (*TP73*) in individuals with sporadic ALS [3]. The researchers further showed that the mutations promoted apoptosis and impaired motor neuron development with in vitro and in vivo functional analyses. *TP73* is part of the p53 family of tumor suppressor transcription factors, which modulate the expression of target genes to affect cell cycle arrest, apoptosis, and cellular differentiation. However, one replication study from the European population among 8230 cases and 9671 controls failed to identify association between rare variants in *TP73* and risk of ALS [4]. Therefore, the genetic role of *TP73* in ALS still needs further replication, especially in cohorts with different ethnic backgrounds.

In this context, we aimed to evaluate the rare protein-coding variants of *TP73* in ALS in the Chinese population. We identified one variant p.P335T which was reported previously, and three rare variants in the same amino acids as the variants reported in previous studies (p.R36Q, p.R78C, and p.R414Q), but did not detect enrichment of *TP73* rare variants in ALS.

## Material and methods

### Participants

Totally 2011 ALS patients of Chinese ancestry were recruited from the Department of Neurology of West China Hospital of Sichuan University. Two specialized neurologists diagnosed the patients according to the El Escorial criteria. All the patients have signed informed consent. The control group of the Taiwanese Schizophrenia Trio Collection was used as controls (*n* = 3298) [5], who were unaffected parents of probands diagnosed with schizophrenia.

### Sequencing

Genomic DNA was extracted from peripheral blood mononuclear cells using standard phenol–chloroform procedures [6]. Whole exome sequencing (WES) was conducted on the Illumina NovaSeq 6000 system following the manufacturer’s instructions. For controls from the Taiwanese Schizophrenia Trio Collection, DNA collected from blood was sequenced on Illumina HiSeq sequencers [5].

### Variant analysis

The rare variants which met the following criteria were analyzed: (1) minor allele frequency (MAF) was lower than 0.01; (2) variants were annotated as missense, splice donor, splice acceptor, start-lost, stop-gained, stop-loss or frameshift substitution using the Ensembl Variant Effect Predictor (VEP) [7]; (3) the variant was either heterozygous or homozygous. Allelic association analysis was performed with standard Fisher’s exact test using default parameters. A Bonferroni-corrected *P* value below 0.05 was considered significant. The summary data of the East Asian population from gnomAD v2.1.1 was used as population control as well.

### Gene-based burden analysis

Gene-based rare variant burden analysis was conducted to evaluate the aggregate association of rare variants in *TP73* with ALS using the optimized sequence kernel association test (SKAT-O, R package) under 100 permutations. Sex and the first three principal components derived from population structure using GCTA v1.93.1 were adjusted. We categorized variants into rare (MAF < 0.01) and ultra-rare variants (MAF < 0.001). For each category, we tested the association for all rare variants and rare damaging variants, which were predicted as damaging by the REVEL tool (score > 0.7) [8].

## Results

We analyzed the rare variants of *TP73* in 2011 patients with ALS of Chinese ancestry. The average age at onset (SD) was 54.32 (11.76) with a sex ratio of 1.45 (male/female: 1190/821) (Additional file 1: Table S1). A total of 24 rare variants (MAF < 0.01) were identified, including 1 stopgain variant and 23 missense variants, among which four were predicted as damaging by REVEL (Additional file 1: Tables S2 and S3). Among the 24 rare variants, 23 were ultra-rare (MAF < 0.001), and nine were absent in the controls and East Asian population from gnomAD (Additional file 1: Table S2). The variant p.P335T identified in our cohort was also reported in the previous study [4], and another three variants (p.R36Q, p.R78C, and p.R414Q) were in the same amino acids as previous variants [4, 9], namely p.R36P, p.R78H, and p.R414W. The clinical data of the patients with the four variants are shown in Table 1. The variant p.R78C had a REVEL score of 0.66 and CADD score of 23.1, suggesting potential deleteriousness (Additional file 1: Table S3). In contrast, the other three variants had relatively lower REVEL scores. No variant was associated with the risk of ALS by Fisher’s exact test (Additional file 1: Table S2), and rare variants at gene level were not enriched in the patients (Table 2). All the patients with the *TP73* rare variants were sporadic. No specific clinical characteristics were observed in the patients with *TP73* rare variants (Additional file 1: Table S4).

**Table 1:** Clinical features of patients with specific variants in *TP73*

Clinical feature	Patient 1	Patient 2	Patient 3	Patient 4	Patient 5
Variant	1:3599665_G_A	1:3599665_G_A	1:3624158_C_T	1:3644710_C_A	1:3647535_G_A
Hgvs_c	c.G107A	c.G107A	c.C232T	c.C1003A	c.G1241A
Hgvs_p	p.R36Q	p.R36Q	p.R78C	p.P335T	p.R414Q
Age at onset (years)	50	62	45	52	46
Sex	F	F	M	M	M
Fasciculation	No	No	No	Yes	Yes
Spasticity	No	No	No	No	No
Drug	No	No	No	No	No
Smoking	Now	No	No	No	No
Drinking	No	No	No	No	No
Sensory	Normal	Normal	Normal	Normal	Normal
Toxin	No	No	No	No	No
Onset site	Proximal lower limb	Proximal upper limb	Proximal upper limb	Proximal upper limb	Proximal upper limb
Onset symptom	Fatigue	Fatigue	Fatigue	Fatigue	Fatigue
Tongue Myoclonus	No	No	No	No	Yes
Tongue atrophy	No	No	Yes	No	Yes
Choking	No	No	Yes	No	No
Dysphagia	No	No	Yes	No	Yes
Dysarthria	No	No	Yes	No	Yes
Pharyngeal reflex	Normal	Normal	Hyporeflexia	Normal	Normal
Family history	No	No	No	No	No

Genomic position was based on GRCh37

*F* female, *M* male

**Table 2:** Enrichment analysis of rare variants in *TP73* in amyotrophic lateral sclerosis

Variant group	MAF < 0.01			MAF < 0.001		
	Case	Control	*P*	Case	Control	*P*
All variants	34	45	0.47	28	34	0.49
Damaging variants	11	20	0.71	10	9	0.32

Rare variant denotes variants with minor allele frequency < 0.01; Ultra-rare variant denotes variants with minor allele frequency < 0.001. Damaging variants denote variants which were predicted as damaging by REVEL (score > 0.7). Case and Control denotes the number of alleles detected in patients and controls

## Discussion

In the current study, we explored the rare variants of *TP73* in a large ALS cohort in the Chinese population. We identified one reported variant p.P335T and another three variants (p.R36Q, p.R78C, and p.R414Q) in the same amino acids as the variants reported in previous studies. However, at gene level rare variants of *TP73* were not enriched in ALS. These results broadened the current mutation spectrum of *TP73* in ALS, and provided a foundation for further research.

*TP73* is a member of the p53 family of transcription factors, and plays an important role in neurogenesis and germ cell maturation [10, 11]. Functional exploration suggested the mutations of *TP73* could cause abnormal differentiation and increase apoptosis in the myoblast differentiation assay, suggesting potential role of *TP73* in ALS. However, the limited sample size in the original study might be prone to false positive [3]. A subsequent replication study in a total of 8230 ALS cases and 9671 controls failed to identify enrichment of rare protein-coding variants in patients with ALS [4]. Therefore, the genetic role of *TP73* in ALS is still questionable.

In the current cohort, one patient carried the same variant p.P335T as reported in the previous study [4]. WES suggested this patient did not carry pathogenic mutations in known ALS-related genes (https://alsod.ac.uk/). This variant is very rare in both the European and East Asian populations (MAF < 0.0001) based on public data from gnomAD. This variant has a Genomic Evolutionary Rate Profiling (GERP) score of 4.79, suggesting high evolutionary constraint. Detection of such a rare variant in patients from different ancestries suggested its potential role in ALS. However, considering the limited sample size of our study and the variant was detected in the control set as well, more evidence from additional cohorts was still necessary. Clinically, the patient carrying p.P335T variant presented weakness in the proximal upper limb and developed ALS at the age of 52 (Table 1).

In addition, another three variants (p.R36Q, p.R78C, and p.R414Q) in the same amino acids as the variants reported in previous studies were identified in our cohort. All the three variants were ultra-rare (MAF < 0.001). WES suggested the patients were free of pathogenic mutations in known ALS genes. Clinically, the four patients carrying the above three variants developed initial presentation with weakness in the proximal upper or lower limb in adulthood (Table 1).

Though several rare variants were detected in the patients only, we did not identify an association between the rare variants and the disease risk at both variant and gene levels. However, the results should be interpreted with caution since the sample size was relatively small. Meanwhile, an association is actually hard to detect for rare variants in case–control designed studies, especially in the context of etiological heterogeneity and incomplete penetrance. Therefore, further replication with a larger sample size was still necessary.

There are also some limitations worth mentioning in the current study. The first is lack of functional evidence. Though we identified a number of rare variants in the patients, we could not identify whether these variants were involved in the pathogenesis of ALS. However, considering the genetic role of *TP73* in ALS was still elusive, more genetic analysis was still necessary before performing further functional exploration. The second is the lack of replication for the identified variants. The current results still need replications in other cohorts in East Asian or Chinese patients, which help to rebut that these variants are just subpopulation specific.

In conclusion, we systematically analyzed the rare variants of *TP73* in ALS of Asian ancestry with association analyses at variant and gene levels. Gene-level burden analysis did not detect enrichment of rare variants in the patients, disproving the genetic role of *TP73* in ALS to some extent. Current results did not support the involvement of *TP73* rare variants in ALS.

## Supplementary Information

**Additional file 1**. Supplementary information about the identified rare variants of TP73.

## Data Availability

All data generated during this study are included in this published article and its supplementary information files. The genotype and phenotype data of the Taiwanese Schizophrenia Trio Collection was applied from dbGap (accession number phs001196.v1.p1).

## Abbreviations

ALS: Amyotrophic lateral sclerosis
SKAT-O: Optimized sequence kernel association test
*TP73*: Tumor protein p73
WES: Whole exome sequencing
MAF: Minor allele frequency
VEP: Variant effect predictor
GERP: Genomic evolutionary rate profiling
