# Supplementary material for: Lack of association of TP73 rare variants with amyotrophic lateral sclerosis in a Chinese cohort
Source: Hum Genomics. 2022 Nov 30;16:63. doi: 10.1186/s40246-022-00437-5 (PMC9710084; doi:10.1186/s40246-022-00437-5)
Supplement: Supplementary file 1 — Additional file 1. Supplementary information about the identified rare variants of TP73. [file 40246_2022_437_MOESM1_ESM.docx]

**Additional file 1: Table S1. Demographic data for the enrolled cohorts.**

| **Group** | **total No.** | **Age at onset (SD)** | **Male/Female ratio** |
| --- | --- | --- | --- |
| ALS cohort | 2011 | 54.32 (11.76) | 1.45 (1190/821) |
| control cohort | 3298 | Not applicable | 1.04 (1681/1617) |

No., the number of individuals; ALS, amyotrophic lateral sclerosis.

**Additional file 1: Table S2. Rare variants of *TP73* identified in patients with amyotrophic lateral sclerosis.**

| **Genomic position** | **rsid** | **annotation** | **hgvs_c** | **hgvs_p** | **gnomAD as control (N=9,977)** | | | **normal control (N=3,298)** | | |
| --- | --- | --- | --- | --- | --- | --- | --- | --- | --- | --- |
|  |  |  |  |  | **control MAF** | **P** | **OR (95% CI)** | **control MAF** | **P** | **OR (95% CI)** |
| 1:3598961 | rs192866394 | missense | c.G32C | p.G11A | 6.82E-5 (1/14654) | 0.384 | 3.64 (0.05-285.40) | 0.00E+0 (0/6596) | 0.379 | Inf (0.04-Inf) |
| 1:3599628 | n.a. | missense | c.C70G | p.P24A | 0 (0/19954) | 0.168 | Inf (0.13-Inf) | 0 (0/6596) | 0.379 | Inf (0.04-Inf) |
| 1:3599665 | rs778557179 | missense | c.G107A | p.R36Q | 3.81E-4 (7/18388) | 0.668 | 1.31 (0.13-6.86) | 4.55E-4 (3/6596) | 1.000 | 1.09 (0.09-9.55) |
| 1:3624158 | rs777997255 | missense | c.C232T | p.R78C | 1.00E-4 (2/19932) | 0.424 | 2.48 (0.04-47.60) | 0 (0/6596) | 0.379 | Inf (0.04-Inf) |
| 1:3624162 | rs746207826 | missense | c.C236T | p.A79V | 0 (0/18372) | 0.180 | Inf (0.12-Inf) | 0 (0/6596) | 0.379 | Inf (0.04-Inf) |
| 1:3624197 | rs965318245 | missense | c.G271A | p.A91T | 0 (0/18354) | 0.180 | Inf (0.12-Inf) | 0 (0/6596) | 0.379 | Inf (0.04-Inf) |
| 1:3624255 | rs372189156 | stopgain | c.C329T | p.S110L | 1.50E-4 (3/19944) | 0.520 | 1.65 (0.03-20.59) | 0 (0/6596) | 0.379 | Inf (0.04-Inf) |
| 1:3640010 | rs776034769 | missense | c.G709A | p.V237M | 5.45E-5 (1/18332) | 0.327 | 4.56 (0.06-356.81) | 1.52E-4 (1/6596) | 1.000 | 1.64 (0.02-128.64) |
| 1:3644710 | rs746096915 | missense | c.C1003A | p.P335T | 5.02E-5 (1/19920) | 0.308 | 4.95 (0.06-387.61) | 0 (0/6596) | 0.379 | Inf (0.04-Inf) |
| 1:3644750 | rs753631424 | missense | c.G1043A | p.R348Q | 0 (0/19926) | 0.168 | Inf (0.13-Inf) | 0 (0/6596) | 0.379 | Inf (0.04-Inf) |
| 1:3645915 | n.a. | missense | c.A1099C | p.I367L | 0 (0/19954) | 0.168 | Inf (0.13-Inf) | 1.52E-4 (1/6596) | 1.000 | 1.64 (0.02-128.64) |
| 1:3645985 | rs1314610892 | missense | c.G1169A | p.R390Q | 0 (0/19944) | 0.168 | Inf (0.13-Inf) | 0 (0/6596) | 0.379 | Inf (0.04-Inf) |
| 1:3646617 | rs1484844561 | missense | c.T1103C | p.V368A | 5.54E-5 (1/18042) | 0.021 | 13.47 (1.08-703.95) | 0 (0/6596) | 0.054 | Inf (0.68-Inf) |
| 1:3646628 | n.a. | missense | c.A1114G | p.M372V | 0 (0/19954) | 0.168 | Inf (0.13-Inf) | 0 (0/6596) | 0.379 | Inf (0.04-Inf) |
| 1:3647519 | rs762610584 | missense | c.C1225T | p.R409C | 0 (0/18102) | 0.182 | Inf (0.12-Inf) | 0 (0/6596) | 0.379 | Inf (0.04-Inf) |
| 1:3647535 | rs761502228 | missense | c.G1241A | p.R414Q | 5.49E-5 (1/18228) | 0.329 | 4.53 (0.06-354.79) | 0 (0/6596) | 0.379 | Inf (0.04-Inf) |
| 1:3647593 | rs574845163 | missense | c.C1301T | p.P434L | 7.05E-4 (14/19850) | 0.131 | 2.12 (0.67-5.87) | 4.55E-4 (3/6596) | 0.091 | 3.28 (0.70-20.30) |
| 1:3647616 | n.a. | missense | c.C1322T | p.P441L | 0 (0/19954) | 0.168 | Inf (0.13-Inf) | 1.52E-4 (1/6596) | 1.000 | 1.64 (0.02-128.64) |
| 1:3648116 | rs770506799 | missense | c.T1427C | p.I476T | 5.44E-5 (1/18390) | 0.327 | 4.57 (0.06-357.94) | 0 (0/6596) | 0.379 | Inf (0.04-Inf) |
| 1:3649360 | rs779902863 | missense | c.G1385A | p.R462Q | 8.72E-4 (17/19500) | 0.343 | 0.29 (0.01-1.82) | 1.67E-3 (11/6596) | 0.038 | 0.15 (0.00-1.03) |
| 1:3649401 | rs141383785 | missense | c.G1426A | p.A476T | 0 (0/19588) | 0.170 | Inf (0.12-Inf) | 0 (0/6596) | 0.379 | Inf (0.04-Inf) |
| 1:3649411 | rs139568604 | missense | c.T1436C | p.L479P | 1.53E-4 (3/19610) | 0.066 | 4.88 (0.65-36.43) | 3.03E-4 (2/6596) | 0.374 | 2.46 (0.28-29.48) |
| 1:3649450 | n.a. | missense | c.G1475A | p.G492D | 0 (0/19954) | 0.168 | Inf (0.13-Inf) | 0 (0/6596) | 0.379 | Inf (0.04-Inf) |
| 1:3649587 | rs571845429 | missense | c.T1612G | p.C538G | 8.51E-5 (1/11748) | 0.445 | 2.92 (0.04-228.92) | 1.52E-4 (1/6596) | 1.000 | 1.64 (0.02-128.64) |

MAF, minor allele frequency; n.a., not available; Variants with MAF < 0.01 were considered rare; P and OR values were obtained using Fisher’s exact test implemented in R 3.6.2 with default parameters; RefSeq accession numbers: NM_001204187 and NM_001126240. Genomic position was based on GRCh37.

**Additional file 1: Table S3. In-silico pathogenicity predictions for rare variants in *TP73*.**

| **Genomic position** | **hgvs_p** | **GERP++** | **REVEL score** | **CADD score** |
| --- | --- | --- | --- | --- |
| 1:3598961 | p.G11A | 3.12 | 0.296 | 6.442 |
| 1:3599628 | p.P24A | 4.74 | 0.699 | 22.3 |
| 1:3599665 | p.R36Q | -4.38 | 0.266 | 0.219 |
| 1:3624158 | p.R78C | 4.44 | 0.662 | 23.1 |
| 1:3624162 | p.A79V | 3.66 | 0.484 | 22.5 |
| 1:3624197 | p.A91T | 2.56 | 0.449 | 15.09 |
| 1:3624255 | p.S110L | 3.68 | 0.618 | 24 |
| 1:3640010 | p.V237M | 0.109 | 0.303 | 14.88 |
| 1:3644710 | p.P335T | 4.79 | 0.496 | 16.9 |
| 1:3644750 | p.R348Q | 4.69 | 0.652 | 26.6 |
| 1:3645915 | p.I367L | 3.17 | 0.292 | 19.71 |
| 1:3645985 | p.R390Q | 4.3 | 0.677 | 22.1 |
| 1:3646617 | p.V368A | -5.03 | 0.273 | 1.333 |
| 1:3646628 | p.M372V | -4.5 | 0.235 | 0.018 |
| 1:3647519 | p.R409C | -1.51 | 0.311 | 0.162 |
| 1:3647535 | p.R414Q | 3.25 | 0.492 | 15.59 |
| 1:3647593 | p.P434L | 5.03 | 0.782 | 25.7 |
| 1:3647616 | p.P441L | 4.22 | 0.382 | 22 |
| 1:3648116 | p.I476T | 5.51 | 0.361 | 22.5 |
| 1:3649360 | p.R462Q | 4.81 | 0.789 | 32 |
| 1:3649401 | p.A476T | 0.73 | 0.275 | 6.844 |
| 1:3649411 | p.L479P | 4.71 | 0.845 | 24.6 |
| 1:3649450 | p.G492D | 4.71 | 0.329 | 22.1 |
| 1:3649587 | p.C538G | 4.53 | 0.76 | 23.3 |

Genomic position was based on GRCh37. GERP, REVEL and CADD score was annotated using VEP.

**Additional file 1: Table S4. Clinical features of patients with rare variants in *TP73*.**

| Patient number | hgvs_c | hgvs_p | AAO | Sex | Sensory | Onset site | Onset symptom | Tongue Myoclonus | Tongue atrophy | Choking | Dysphagia | Dysarthria | Pharyngeal reflex |
| --- | --- | --- | --- | --- | --- | --- | --- | --- | --- | --- | --- | --- | --- |
| 6 | c.G32C | p.G11A | 34 | F | Normal | proximal upper limb | fatigue | No | No | Yes | Yes | Yes | Normal |
| 7 | c.C70G | p.P24A | 33 | M | Normal | proximal upper limb | fatigue | No | Yes | No | No | Yes | Normal |
| 8 | c.C236T | p.A79V | 34 | F | Normal | proximal upper limb | fatigue | Yes | Yes | Yes | Yes | Yes | Hyporeflexia |
| 9 | c.G271A | p.A91T | 66 | M | Impaired | distal lower limb | fatigue | Yes | Yes | Yes | Yes | Yes | Hyporeflexia |
| 10 | c.C329T | p.S110L | 53 | F | Normal | distal upper limb | fatigue | Yes | Yes | Yes | Yes | Yes | Hyporeflexia |
| 11 | c.G709A | p.V237M | 66 | F | Normal | bulbar | dysarthria | Yes | Yes | Yes | No | Yes | Normal |
| 12 | c.G1043A | p.R348Q | 47 | M | Impaired | proximal upper limb | fatigue | Yes | No | Yes | Yes | Yes | Hyperreflexia |
| 13 | c.A1099C | p.I367L | 50 | F | Normal | proximal lower limb | fatigue | Yes | Yes | Yes | No | Yes | Normal |
| 14 | c.G1169A | p.R390Q | 66 | M | Normal | proximal upper limb | fatigue | No | No | No | No | No | Normal |
| 15 | c.T1103C | p.V368A | 47 | M | Normal | proximal upper limb | fatigue | Yes | Yes | Yes | Yes | Yes | Hyporeflexia |
| 16 | c.T1103C | p.V368A | 48 | M | Normal | proximal upper limb | fatigue | Yes | Yes | Yes | No | Yes | Normal |
| 17 | c.T1103C | p.V368A | 23 | F | Normal | proximal upper limb | fatigue | No | No | No | No | No | Hyperreflexia |
| 18 | c.A1114G | p.M372V | 41 | M | Impaired | proximal upper limb | fatigue | Yes | Yes | Yes | Yes | Yes | Normal |
| 19 | c.C1225T | p.R409C | 61 | F | Normal | proximal lower limb | fatigue | Yes | Yes | No | No | Yes | Normal |
| 20 | c.C1301T | p.P434L | 50 | M | Normal | distal upper limb | fatigue | No | No | No | No | No | Normal |
| 21 | c.C1301T | p.P434L | 43 | F | Normal | proximal upper limb | fatigue | Yes | Yes | No | No | No | Normal |
| 22 | c.C1301T | p.P434L | 39 | M | Normal | proximal lower limb | fatigue | No | No | No | No | No | Hyporeflexia |
| 23 | c.C1301T | p.P434L | 39 | F | Normal | proximal lower limb | fatigue | No | No | No | No | No | Normal |
| 24 | c.C1301T | p.P434L | 44 | F | Normal | proximal lower limb | fatigue | Yes | Yes | No | No | No | Hyperreflexia |
| 25 | c.C1301T | p.P434L | 54 | M | Normal | proximal upper limb | fatigue | Yes | No | Yes | No | Yes | Normal |
| 26 | c.C1322T | p.P441L | 42 | M | Normal | bulbar | choking | Yes | Yes | Yes | Yes | Yes | Normal |
| 27 | c.T1427C | p.I476T | 45 | F | Normal | bulbar | dysarthria | Yes | Yes | Yes | Yes | Yes | Normal |
| 28 | c.G1385A | p.R462Q | 42 | M | Normal | proximal upper limb | fatigue | No | No | No | No | No | Normal |
| 29 | c.G1426A | p.A476T | 52 | F | Normal | proximal upper limb | fatigue | No | No | No | No | No | Normal |
| 30 | c.T1436C | p.L479P | 57 | F | Normal | proximal lower limb | fatigue | Yes | Yes | Yes | No | Yes | Normal |
| 31 | c.T1436C | p.L479P | 54 | M | Normal | distal lower limb | fatigue | No | No | Yes | No | No | Normal |
| 32 | c.T1436C | p.L479P | 68 | F | Normal | proximal upper limb | fatigue | Yes | Yes | Yes | Yes | Yes | Normal |
| 33 | c.G1475A | p.G492D | 40 | F | Normal | proximal upper limb | fatigue | No | No | No | No | No | Normal |
| 34 | c.T1612G | p.C538G | 34 | F | Normal | proximal upper limb | fatigue | Yes | Yes | Yes | Yes | Yes | Normal |

F, female; M, male; AAO, age at onset.
